# Supplementary material for: Resolving the full spectrum of human genome variation using Linked-Reads
Source: Genome Res. 2019 Apr;29(4):635–45. doi: 10.1101/gr.234443.118 (PMC6442396; doi:10.1101/gr.234443.118)
Supplement: Supplemental Material [file supp_gr.234443.118_Supplemental_longranger-2.2.zip › longranger-2.2.tar.bz2!/lr2.2-public/lib/go/src/code.google.com/vitess/web/vtctld2/src/app/workflows/workflow.component.html]

remove
forward
check
{{workflow.name}}

{{workflow.progressMsg}}

Start
Stop
Delete

Last Change: {{getTime()}}

{{workflow.message}}

Log

{{workflow.log}}

{{action.name}}
